# Microbes a Tool for the Remediation of Organotin Pollution Determined by Static Headspace Gas Chromatography-Mass Spectrometry

**DOI:** 10.3390/molecules23030627

**Published:** 2018-03-10

**Authors:** Christopher Finnegan, David Ryan, Anne-Marie Enright, Guiomar Garcia-Cabellos

**Affiliations:** EnviroCORE, Department of Science and Health, IT Carlow, Kilkenny Road, Co., R93 V960 Carlow, Ireland; David.Ryan@itcarlow.ie (D.R.); Ann-Marie.Enright@itcarlow.ie (A.-M.E.); Guiomar.Garcia-Cabellos@itcarlow.ie (G.G.-C.)

**Keywords:** bioremediation, gas chromatography, green chemistry, mass spectrometry, microcosm, organotin analysis, static headspace, tributyltin (TBT)

## Abstract

Tributyltin (TBT) is one of the most toxic anthropogenic compounds introduced into the marine environment. Despite its global ban in 2008, TBT is still a problem of great concern due to its high affinity for particulate matter, providing a direct and potentially persistent route of entry into benthic sediments. Bioremediation strategies may constitute an alternative approach to conventional physicochemical methods, benefiting from the microorganism’s potential to metabolize anthropogenic compounds. In this work, a simple, precise and accurate static headspace gas chromatography method was developed to investigate the ability of TBT degrading microbes in sedimentary microcosms over a period of 120 days. The proposed method was validated for linearity, repeatability, accuracy, specificity, limit of detection and limit of quantification. The method was subsequently successfully applied for the detection and quantification of TBT and degradation compounds in sediment samples on day 0, 30, 60, 90 and 120 of the experiment employing the principles of green chemistry. On day 120 the concentration of TBT remaining in the microcosms ranged between 91.91 ng/g wet wt for the least effective microbial inoculant to 52.73 ng/g wet wt for the most effective microbial inoculant from a starting concentration of 100 ng/g wet wt.

## 1. Introduction

Organotin compounds (OTCs), most notably tributyltin (TBT), have been extensively employed in a variety of industrial products, such as antifouling paints for marine crafts, wood preservatives, biocides, and plastic stabilizers [1,2,3]. Amongst OTCs, a great deal of research has indicated that TBT is one of the most toxic compounds deliberately introduced into the to the aquatic ecosystem. TBT can be described as an effective endocrine disruptor which also exhibits immunotoxin and genotoxic capabilities towards a huge variety of organisms, extending from bacteria to human [2,4]. TBT is persistent in the environment due to its half-life in sediment which can range from 6 months to 8.7 years [5]. TBT binds strongly to suspended materials such as minute organic materials or inorganic sediments due to several factors: (1) hydrophobic forces; (2) high specific gravity (near 1.2 kg/L at “20 °C”; (3) low solubility (<10 mg/L at 20 °C and pH 7.0); (4) octanol-water partition coefficient (logK_ow_) of 3.21 to 4.4 at pH values of 5.8 to 8 [3,6]. The adsorption of TBT to sediments is reversible, thus contaminated sediments can act as a temporary sink and a long-term source of contamination to the overlying water column [6,7]. Therefore, TBT contamination can be found in most busy harbors and shipping lanes even within regulated nations and high concentrations of TBT (36,292 ng Sn/g) in surface sediments are still observed in places where heavy ship-building activities exist [8,9,10,11].

**Figure 1:**
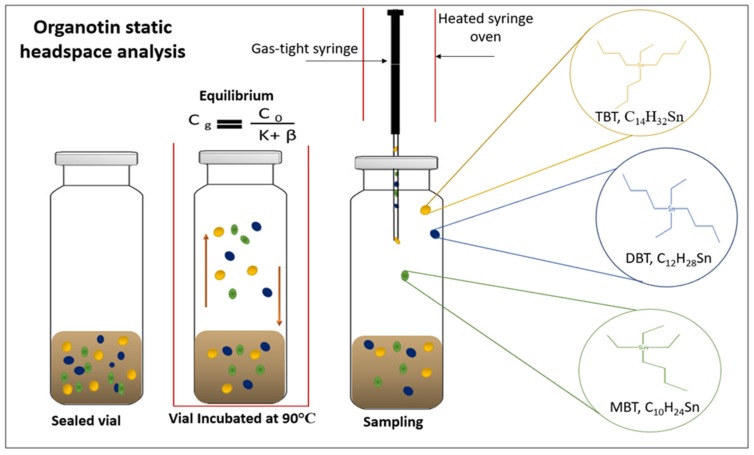
Simple representation of static headspace analysis of organotin compounds TBT, DBT and MBT from sediment. The vapor-phase of the extraction is represented illustrating the equilibration of analytes between a non-volatile solid phase, after heating and agitation of the sample the equilibrium (Cg = (Co)/(k + β) ((Cg) the concentration of the analyte, (Co) analyte concentration, (K) the partition coefficient, (β) volume ratio) [23], of the compounds is reached and the vapor phase mixture which contains fewer interfering compounds is transferred to the GC for analysis.

Quantitative analytical methods have been developed during the last decade to monitor the levels of TBT in sediment which generally involves gas chromatography (GC) with a selective and sensitive detection method [12,13,14,15]. This approach involves several stages which depend on the physicochemical properties of the analytes and the matrix environment [15,16]. Extraction of the OTCs from the sample matrix is a fundamental stage for the analysis. This can be incomplete as OTCs are strongly bound to particular matter and as a result current methodology often fails in the accurate quantitative determination of monobutyltin (MBT) and to a lesser extent dibutyltin (DBT). The most widely used technique is liquid–liquid extraction i.e., leaching under acidic conditions with acetic acid or HCl and a medium to low polarity solvent (e.g., dichloromethane, *n*-hexane, or tetrahydrofuran) [10,15,17]. The principal drawbacks associated with liquid–liquid extraction include: (1) time required for sample pre-treatment; (2) number of analytical steps; (3) potential losses of analytes; (4) health hazard in handing large amounts of volatile organic solvents [18]. An alternative green chemistry method is the use of static headspace (SHS) analyses as this technique offers numerous advantages such as reduced solvent use, higher recoveries, good repeatability, simplifying of the sample preparation and avoids possible interference from complex matrixes such as sediment [19,20,21]. Headspace analysis is generally defined as a vapor-phase extraction of analytes between a non-volatile liquid or solid phase. The vapor phase mixture contains fewer interfering compounds and is transferred to the GC for analysis (Figure 1). Several well-documented reviews have been published on the principles and instrumentation of headspace and SHS [19,20,21,22].

Bioremediation is recognized as a major process for TBT removal and numerous studies have taken place involving the isolation and characterization of TBT resistant and degrading microbes which including the following genera, *Klebsiella, Alcaligenes*, *Aeromonas*, *Enterobacter*, *Bacillus*, *Pseudomonas* and *Citrobacter* [3,24,25,26,27,28]. A limited number of bench scale laboratory investigations have taken place to study the behavior of microbes under various concentrations of TBT which included microcosm studies [3,29,30,31], and media [5,25,26,27,28,32], investigations. However, to gain information on the mechanistic reactions and to investigate TBT degradation rates to DBT and MBT by isolated microbes, it is essential that further investigations take place under controlled environmental microcosm and mesocosm conditions.

The present study is a continuation of previous work by the authors, whereby TBT resistant and TBT utilizing microbes were isolated from a variety of sediments and soil samples using TBT containing media. Candidate microbial isolates were identified via 16S rRNA analysis and their TBT degrading activity was evaluated via growth plate assays and further confirmed by gas chromatography ion trap mass detection [15]. The aim of this study is to further evaluate the microbial ability to degrade TBT in sediment. Thus, microcosms were created in the laboratory containing TBT spiked sediment in which each isolate from the previous study were inoculated with and sediment samples were analyzed on day 0, 30, 60, 90 and 120 for TBT and the biodegradation intermediates dibutyltin (DBT), and monobutyltin (MBT). Due to the complexity of the sediment matrix, a robust analytical method was development to establish the efficiency of the microbes in the remediation of TBT contaminated sediments.

## 2. Results and Discussion

### 2.1. System Suitability

The suitability of a SHS-MS method for the qualitative and quantitative analysis of OTCs in sediment samples was assessed, optimized and validated in terms of key analytical parameters including separation, detection, extraction, accuracy, linearity, recovery and sensitivity. The method was then employed to determine the ability of the six microbes to degrade TBT under microcosm conditions. Selected ion monitoring (SIM) mode was employed for the chemical analysis of the sediment samples extracted from the microcosm due to its lower sensitivity and its ability to eliminate difficult matrix interferences in sediment. Thus, for each organotin compound of interest, three ions not affected by interferences were monitored to provide good specificity, using the most abundant ion for quantification: TPrT 149, 179, 207 and MBT 149, 179, 235 *m*/*z*, DBT 149, 207, 235 and TBT 207, 263, 291 *m*/*z* [33,34,35]. Although the headspace mode has scarcely been used for speciation of OTCs, in this mode the gas phase containing the volatile substances is injected into the GC column and analyzed without the extraction of non-volatile interfering compounds [23].

### 2.2. Optimisation of Static Headspace Conditions

For method optimization, temperature and time was investigated to establish the equilibrium between the sample matrix and the gas phase. In equilibrium, a relationship between the gas and the sample phase (sediment) concentrations for volatile compounds is expressed as the partition coefficient (K) [20]. This parameter represents the ratio of the analyte’s concentration in the two phases: sample phase (cS) and the gas phase (cG): K = cS/cG. Under given conditions, K is constant, thus the concentration in the headspace is proportional to the original concentration (co). The value of K will be dependent on both the compound and the sample matrix and it will also be strongly affected by temperature. Following the basic chromatographic rules, the obtained peak area of the analyte will be directly proportional its cG and, therefore, to its concentration in the original sample (co) [21,23,36].

#### Temperature and Time Effect

**Figure 2:**
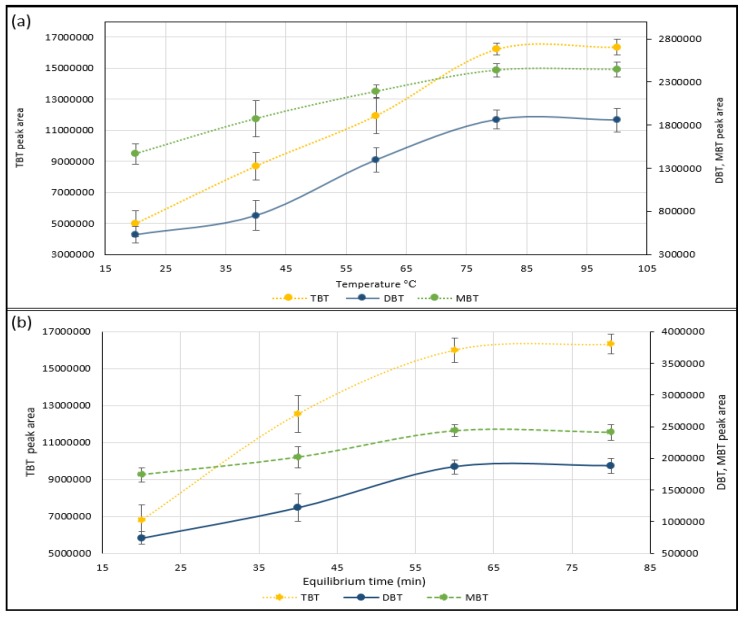
Optimization of the parameters affecting the extraction of organotin compounds TBT, DBT and MBT from 20 ng/g spiked sediment samples by repeated measures (*n* = 3) against peak area; (**a**) reaction temperature between 20 °C and 100 °C was investigated for greater extraction yields of TBT, DBT and MBT in 20 °C increments; (**b**) reaction time was investigated to achieve a constant state of equilibrium for TBT, DBT and MBT from peak area against time profiles 20, 40, 60 and 80 min at 90 °C. Error bars represent the standard of the mean (SD±).

Temperature, time, and agitation can be used to improve the transfer of volatile analytes (TBT, DBT and MBT) from the sample matrix into the headspace of the vial. Adjusting the temperature of the sample will change the solubility of the analyte in the sample matrix and can be used to drive the equilibrium in favor of the gas phase [19,20,23]. The equilibrium temperature between 20 °C and 100 °C was investigated for greater extraction yields of TBT, DBT and MBT in 20 °C increments, against peak area by repeated measures (*n* = 3) of 20 ng/g wet wt spiked sediment samples (Figure 2). Results show that increasing the temperature produced an improvement in the analytes yield (peak area) from the samples matrix to headspace due to analyte volatility enhancement and therefore this is a suitable method to improve extraction efficiency. Figure 2 also shows that the equilibrium of TBT, DBT and MBT was reached between 80 °C and 100 °C. As a result, 90 °C was selected for improving the simultaneous extraction of the OTCs. A further increase in temperature was not attempted for safety reasons due to vial pressure and a temperature of 90 °C was selected to limit water vapor injection on the column. Sufficient time was also considered to achieve a constant state of equilibrium and maintaining the equilibrium temperature gives good reproducibility. At shorter equilibration times, the sample does not reach equilibrium, and partitioning between the solid phase and the gas phase remains incomplete. The effect of equilibration times for TBT, DBT and MBT was established from peak area against time profiles 20, 40, 60 and 80 min by repeated measurements (*n* = 3) of spiked sediment samples (20 ng/g) at 90 °C. The area for TBT, DBT and MBT increased progressively up to 60 min and thereafter the equilibrium is attained (Figure 2). Therefore 65 min was selected as the operating condition because it allows the multianalyte extraction of OTCs in an equilibrium state.

### 2.3. Method Validation

The method was validated using optimized conditions involving a procedure that suggests that the method yields adequate consistency, precision and accuracy. Therefore, the measurements of repeatability, sensibility, linearity and detection limits were investigated. 

#### 2.3.1. Linearity and Precision

The linearity assessment determines the ability of the procedure to obtain test results which are proportional to the concentration of the analyte in the sample within a given range. Thus, the linearity of the method was studied by preparing six-point calibration curves of matrix matched standards of three concentration levels 0–10, (0, 2, 4, 6, 8, and 10 ng/g wet wt), 0–25, (0, 5, 10, 15, 20, and 25 ng/g wet wt) and 0–50 (0, 10, 20, 30, 40, and 50 ng/g wet wt) ng/g wet wt of TBT, DBT, MBT and analyzed in triplicate per level. The graphs were plotted using peak area of each component on the y-axis and the corresponding concentration on the x-axis. The peak area of TBT, DBT and MBT were linear with respect to the concentrations of the analytes. A good correlation was found between the observed peak area ratios (*y*) and the theoretical concentration (*x*). Least-squares regression analysis provided typical regression lines: *y* = 787,443*x* − 1 × 10^6^ (R^2^ = 0.995) for TBT, *y* = 92,630*x* − 8447.2 (R^2^ = 0.999) for DBT and *y* = 122,012*x* + 931.25 (R^2^ = 0.998) for MBT. Precision is the measure of the degree of repeatability of an analytical method under normal operation and is normally expressed as the percent relative standard deviation (%RSD) for a statistically significant number of samples. Therefore, the precision of the method was established by carrying out the analysis for ten consecutive replicates of standards to obtain the retention time (RT) of the analyte of interest and RSD%. Results show suitable repeatability and the mean RT for the identification of the ethylated OTCs are, TPrT 7.26, MBT 9.73 DBT 11.89 and TBT 13.74 min respectively with a standard deviation of 0.015, 0.031, 0.029, 0.028 respectively and all analytes have less than a 0.1% RSD.

#### 2.3.2. Accuracy & Sensitivity

The accuracy of the developed method, expressed as percentage recovery was studied using spiked (TPrT, TBT, DBT and MBT) sediment samples of various concentrations (*n* = 3) (between 5 and 60 ng/g wet wt). The mean results of recoveries obtained were in the range of 94%–103% with RSDs below 10% for all OTCs under investigation indicating that good recoveries were obtained. However, for a further assessment of this method, the use of certified reference materials such as PACS-2 harbor sediment, (National Research Council of Canada) or BCR 462R (Institute for Reference Materials of the European Commission) should be applied. The sensitivity of the method was demonstrated by establishing the limits of detection (LOD) and quantitation (LOQ) for TBT, DBT and MBT in spiked sediments. The detection and quantification limits were determined experimentally as the lowest concentration giving a chromatographic peak three times the signal/noise ratio and ten times the signal/noise ratio, respectively by auto-integration of the instrument [35,37]. The LOD for the OTCs, was 0.7, 0.1, 0.4, and 0.1 ng/g wet wt and the LOQ was 2.3, 0.3, 1.3 and 0.3 ng/g wet wt for TPrT, TBT, DBT, and MBT respectively which was acceptable for the test samples.

### 2.4. Microcosm Chemical Analysis 

Biodegradation as an alternative to physicochemical remediation utilize microbes as the major pathway for the removal of TBT contamination in sediment through degradation of TBT to its less toxic compounds DBT and MBT. Even though the environment can self-recover from TBT contamination by indigenous microbes, the process can be slow without intervention. Earlier studies resulted in the isolation of 6 microbes that can utilize TBT as a sole carbon source in mineral salt medium and can degrade TBT to its less toxic compounds. In brief the results showed a decrease of TBT in liquid samples ranging from 22% to 70% and the formation of degrading products DBT and MBT [15]. Thus, it is relevant to investigate these microbes (C3, C6, C7, C18, C21 and C22) under conditions that better mimic environmental conditions.

**Figure 3:**
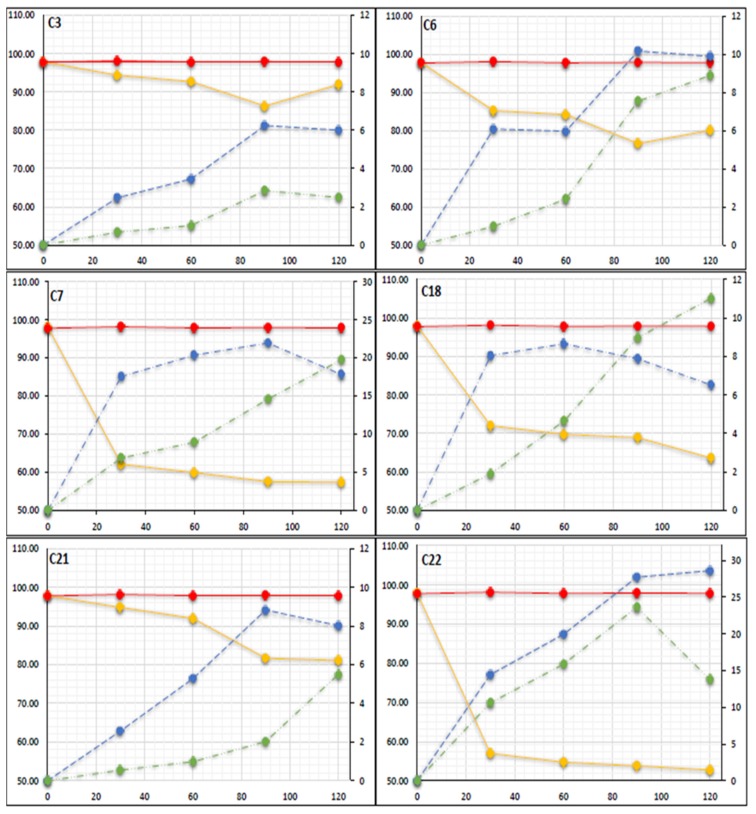
GC-MS quantification analysis of TBT (100 ng/g) spiked sedimentary microcosms inoculated with microbes; C3, C6, C7, C18, C21 and C22 over a 120-day period (Data presented as mean by repeated measures (*n* = 3)). Dual y-axis ng/g, left y-axis represents TBT (gold) degrading and right y-axis represents the formation of DBT (blue) and MBT (green) red plot represents TBT in control microcosms x-axis represents sampling days.

In the present study, the quantification of TBT (100 ng/g) degradation in spiked sediment microcosm inoculated with microbes; C3, C6, C7, C18, C21 and C22 over a 120-day period was carried out by matrix matched calibration curves using the external standard method (Figure 3). From the results it can be seen that the microbes degraded TBT between 5.2% (minimum) and 38% (maximum) during the first 30 days showing a degradation rate of between 0.174*–*1.263 ng/g/day. Results show that from days 30 to 90 despite the average degrading rate of TBT slowing down to between 0.03–0.21 ng/g/day, the formation of the less toxic degrading products DBT and MBT were detectable at concentrations between 2.4*–*27.7 and 0.5*–*23.6 ng/g wet wt respectively. On day 120 the concentration of TBT remaining in the microcosms ranged between 91.91 ng/g wet wt for the least effective microbial inoculant to 52.73 ng/g wet wt for the most effective microbial inoculant. Thus, the microbes under investigation, C3, C6, C7, C18, C21 and C22 degraded TBT by a minimum of 8% and a maximum of 47%. 

In a similar study the degradation of TBT was investigated in microcosms from sediment collected from the Mekong River using the indigenous microbial population [30]. The initial concentration of TBT ranged between 1.0 to 1.4 μg/g dry wt. After 150 days the results showed a maximum decrease of TBT of 57% compared to controlled autoclaved sediments were TBT remained constant thus the results suggested that microorganisms in the Mekong River sediment have a high ability to degrade TBT. In a closer related study, a microcosm experiment, using contaminated sediment from Ria de Aveiro, Portugal, was carried out in order to investigate the ability of *Aeromonas molluscorum* Av27, to bioremediate TBT alone and in association with the indigenous bacterial community. After 150 days, 28% of the TBT was degraded into DBT and MBT and results showed a higher TBT degradation occurred when the concentration of AV27 was increased [3]. However, a direct comparison of microcosm experiments cannot be made due to several reasons such as, TBT absorption varies depending on sediment characteristics such as the granulometry, amount of organic matter [3] and the microcosms experimental design (container wall, environmental conditions, days of exposure, sediment analysis). It is also important to mention bioavailability can reduce biodegradation performance of microbes in aged contamination [38]. Nevertheless, these studies and other suggests that bioremediation is effective for TBT contaminated sediment.

**Figure 4:**
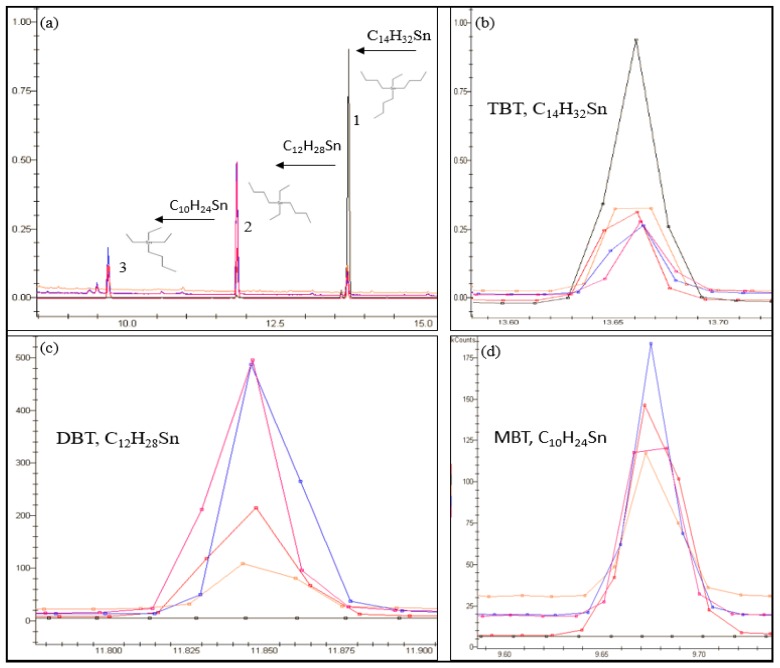
An overlay of multiple chromatograms in selected ion monitoring (SIM) (149, 179, 207, 235, 263, 291 *m*/*z*) (detector response, K count (*y*-axis) against retention time minutes (*x*-axis)), showing sediment samples analyzed from the microcosm inoculated with C22 on day 0 (black), 30 (orange), 60 (red), 90 (blue) and 120 (pink). Image (**a**,**b**) demonstrates the microbes (C22) ability to degrade TBT (peak 1) to the less toxic species DBT (peak 2) by a sequential loss of an alkyl group and further to MBT peak (3). Images (**c**,**d**) emphasizes on the formation of DBT and MBT over the 120 day period from the overlaid chromatograms.

Several mechanisms have been proposed for the survival of bacteria in the presence of TBT such as an efflux pump (efflux of TBT outside the isolated cell) and adsorption and biosorption [3,27,28]. From Figure 3 it can be seen that DBT was the primary degradation product at concentration between 3.45–28.6 ng/g wet wt and on days 90 to 120 and MBT was detectable at concentrations between 2 and 23 ng/g wet wt, concluding therefore that TBT was degraded in a stepwise manner. Thus, the microcosm investigation and additional studies indicated that microbes C3, C6, C7, C18, C21 and C22 can degrade TBT, by a dealkylation mechanism [24,26,27,38], to less toxic species by a sequential loss of an alkyl group by the following method: TBT (C_12_H_27_Sn^+^) > DBT (C_8_H_18_Sn^2+^) > MBT (C_4_H_9_Sn^3+^) over time (Figure 4). Statistical analysis shows that there is a significant difference (*p* < 0.05) between the degradation of TBT in each microcosm and that higher TBT degradation occurred in microcosms inoculated with isolates C7 and C22 (Figure 3) with an initial rate of 1.26, and 1.493 ng/g/day respectively. The results also showed a final concentration in the microcosms inoculated with C7 and C22 of, 57.38, 52.73 for TBT, 17.81, 28.5 for DBT and 19.75, 13.8 ng/g wet wt for MBT respectively suggesting that these two microbes have a particularly high potential to degrade TBT.

The rates of TBT degradation may be influenced by several biotic and abiotic factors, for instance, the nature and density of the microbial population, TBT solubility, dissolved/suspended organic matter, pH, salinity, temperature, and light [24]. Unfortunately, generally on day 90 TBT degradation reduced to a rate of 0.02 to 0.34 ng/g/day. This could be due to several reasons including, poor microbial activates due to the lack of essential nutrients resulting in lower growth rates. One possible method to enhance and accelerate the degradation of TBT in sediment further would be an additional inoculation of microbes on day 90. Another possible solution may be the addition of nutrients through an organic substrate (compost, straw) or electron acceptors (i.e.*,* nitrogen, oxygen, carbon and phosphorus) to further accelerate the degradation of TBT [38,39], and to provide protection to microbial cells from TBT stress as they can utilize other carbon sources [28].

## 3. Materials and Methods

### 3.1. Chemicals and Reagents

All chemicals and reagents were purchased from Sigma-Aldrich (Vale Road, Arklow, Wicklow, Ireland) unless otherwise stated. Tributyltin chloride (TBT) (95%), dibutyltin dichloride (DBT) (97%), butyltin trichloride (MBT) (95%) and tripropyltin chloride (TPrT) standards and individual stock solutions were prepared at a concentration of 1000 µg/L in methanol (99.7%, GC grade). From these, intermediate working standards, containing the three analytes were prepared for calibration purposes. All stock solutions and working standards in methanol were stored in darkness at 4 °C and the final diluted working standards were freshly prepared immediately prior to use. Sodium tetraethylborate (NaBEt4) (97%) (Fisher Scientific, Dublin, Ireland) is commercially available in bottles of 1 g thus a 20% (*w*/*v*) stock solution was prepared by dissolving the entire contents of a 1 g bottle of the reagent in 5 mL of tetrahydrofuran (THF) directly. From this stock solution a 5% (*w*/*v*) working standard was freshly prepared for use. Sodium acetate (82 g/L in deionized H2O) and acetic acid was used to adjust the pH. Tropolone (98%) (2-Hydroxy-2,4,6-cycloheptatrien) was prepared in isooctane (99%) (2,2,4-trimetylpentane) (Fisher Scientific, Dublin, Ireland), to increase the extraction of mono- and disubstituted.

### 3.2. TBT Degrading Isolates

Bacterial isolates, C3, C6, C7, C18, C21 and C22, were previously characterized and identified by the authors and were deposited in the GenBank database under the accession numbers KX881904–KX881909. In brief bacterial isolates C3, C7, C18 and C21 were isolated from several soil samples taken from the traverser pit located on Dinish Island in Bear Heaven Ireland and isolates C6 and C22 came from sediment samples taken from Cork harbor Ireland. Isolates C6, C18, C21 and C3 are members of the class Beta-Proteobacteria, genus Achromobacter while Isolates C7 and C22 are members of the class Gamma-Proteobacteria, genus Enterobacteriales [15].

### 3.3. Microcosm Experiment Setup

**Figure 5:**
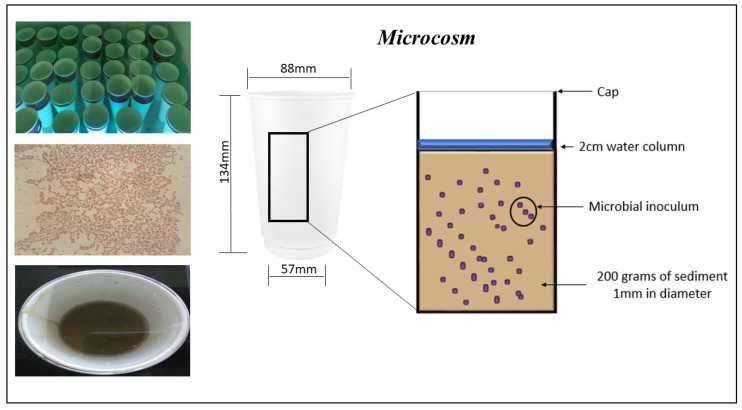
Microcosm set up to establish the efficacy of microbial isolates C3, C6, C7, C18, C21 and C22. Each microcosm includes 200 g of spiked sediment with 100 ng/g of tributyltin chloride and a 2-cm water column which was aspeticaly inoculated with 10 mL of an overnight culture in nutrient broth of an individual isolate and sealed.

Sediment samples were collected from Courtown beach latitude 52°38′53.4″ north and longitude 6°13′33.5″ west Co. Wexford, Ireland where no known pollution of TBT has taken place. The sediment was autoclaved for 40 min at 121 °C to destroy any indigenous bacterial strains. Granulometry of the sediment can affect TBT absorption and so sediment was sieved to a 1mm diameter to allow comparison between samples. Sediment was spiked with 100 ng/g of tributyltin chloride in a methanol solution and was air dried for 1 week and homogenized by mixing twice daily. Microcosms were prepared in containers (95% cardboard and 5% polyethylene) previously washed with ethanol, antibacterial agent and placed under UV for sterility and stored in sterile bag. Microcosm conditions were established containing 200 g of spiked sediment (100 ng/g of tributyltin chloride) and a 2 cm water column (Figure 5).

Each microcosm was aspeticaly inoculated with 10 mL of an overnight culture in nutrient broth of an individual bacterial isolate C3, C6, C7, C18, C21 and C22 respectively and sealed. Triplicates of each condition were prepared which included non-inoculated microcosms. All microcosm experiments were incubated in the dark at room temperature. At days 0, 30, 60, 90 and 120 sediment samples were homogenized and withdrawn by taking 5 g from each microcosm under the same conditions to a 15 g total, aspetic, and stored in a polypropylene bag at −70 °C for subsequent chemical analysis.

### 3.4. Organotin Chemical Analysis

A 1 g subsample of a 15 g sediment extract was weighed into a 20 mL headspace vial followed by the addition of a 1 mL sodium acetate/acetic buffer (pH 4.7) and 1 mL of a 0.1% tropolone solution in isooctane. Additionally, 5 µL of a 10 µg/L tripropyltin chloride solution in methanol was added to each sample as a recovery internal standard (RIS). Samples were hermetically closed using a PTFE coated septum. Derivatization was performed by adding (via syringe), 200 µL of a freshly prepared 5% sodium tetraethylborate solution (NaBEt4) at 3 min intervals for 15 min for a total addition of 1 mL under continuous agitation by sonication for the ethylation of OTCs present in the sample. The vials were then placed in the autosampler for headspace extraction whereby samples were further agitated by oscillation under optimized conditions (Table 1). A heated gas-tight syringe was then used to sample the vapors within the vial (static headspace). Each sample was carried out in triplicate and blank samples were analyzed with every batch to ensure no contamination was present.

### 3.5. GC-MS Operating Conditions

The separation and detection of organotin species TBT, DBT and MBT was performed by a Varian 450-GC, ion trap 220-MS system, with CombiPAL auto sampler (Varian Inc., Walnut Creek, CA, USA) (Table 1). In brief, separation was carried out on a non-polar capillary column and helium of high purity was employed as the carrier gas, at a flow rate of 1 mL/min. The temperature program was as follows: 50 °C for 4 min and the temperature was then increased by 10°/min to 300 °C. The MS-detector was operated in full scan mode in the range of 40*–*650 mass-to-charge ratio (*m*/*z*) to determine the appropriate masses for selected ion monitoring (SIM). 

### 3.6. Data Analysis

Chromatographic data processing was carried out using MS Workstation (6.0). Additionally two-way analysis of variance (ANOVA, Two-Factor) was used to analyze the data among the bacterial isolates regarding TBT degradation in the established microcosms. The data was expressed as mean standard deviation with each assay conducted in triplicate (*n* = 3). The significance level was set at α = 0.05 for all statistical test. 

## 4. Conclusions

The static headspace method presented in this study has shown to be effective at determining the efficacy of the microbes under investigation and incorporates the most important requirements for “greener” sample preparation techniques such as, less organic solvents and less sample preparation, thus diminishing the negative effects of analytical chemistry on the environment. Using static headspace, volatile or (semi-) volatile analytes can be injected selectively into GC, leaving the non-volatile compounds in the headspace vial. Therefore, only volatile molecules are being transferred to the chromatographic system which leads to an overall improvement in analytical performance.

The results presented have confirmed the ability of the selected microbes C3, C6, C7, C18, C21 and C22 to convert the toxic compound TBT into less or non toxic products DBT and MBT. In particular, isolates C7 (KX881905) and C22 (KX881904) which showed a 42.6%, and 47.2% TBT reduction respectively of the original spiked sediment, show the greatest potential for utilization in a bioremediation of TBT in contaminated sites. Thus, using nature-based solutions i.e., bioremediation is a viable solution for the removal of TBT in sediment, this process has advantages over physicochemical approaches as this method produces no waste, it has a lower cost of operations and reduces health and ecological effects and can be formed in situ without disturbing the environment.

## Figures and Tables

**Table 1:** GC/MS conditions used for the analysis of organotin compounds.

Instrument Conditions	
Carrier gas	Helium (99.999%)
Flow rate	1 mL/min
Injector port temperature	280 °C
Column	5% biphenyl and 95% dimethylpolysiloxane 30 m length × 0.25 mm internal diameter × 0.25 μm film thickness.
Temperature program	
Initial temperature	50 °C for 1 min
Ramp	10°/min
Final temperature	300 °C held for 4 min
Split ratio	Splitless
Detector temperature	280 °C
Injection conditions	Headspace
Thermosatatting temperature	90 °C
Thermosatatting time	65 min
Agitation speed	350 rpm
Agitation on time	10 s
Agitation off time	2 s
Syringe temperature	95 °C
Syringe volume	2.5 mL
Injection volume	1.00 mL
Injection fill speed	100 µL/s
Injection speed	200 µL/s
Syringe flushing time	4 min at 1 bar pressure
Mass spectrometer conditions	
Mode	Electron ionisation (70 Ev)
Acquisition mode	Selected ion monitoring
